# The Diagnosis, Pathology, and Treatment of the Diseases of the Chest

**Published:** 1850-10

**Authors:** 


*The Diagnosis, Pathology, and Treatment of the Diseases of the Chest.* By W. W. Gerhard, M. D., Lecturer on Clinical Medicine to the University of Pennsylvania, one of the Physicians to the Pennsylvania Hospital. *Third Edition, Revised and Enlarged.* Philadelphia: Ed. Barrington & Geo. D. Haswell. 1850.

Those who acknowledge the importance of physical diagnosis in diseases of the chest—and who does not?—will hail with peculiar pleasure this new edition of a popular work from the hands of a "master in Israel." The name of its author is closely associated with this subject as one who has done much in its elucidation. This edition contains a variety of new matter, besides a more full development of many portions of the former one. A statement of the effects of cod liver oil in the treatment of consumption is among the novelties introduced. In regard to the question whether cod liver oil ever cures phthisis, the author remarks, that he has "never yet met with a case in which the physical signs, as well as the general symptoms, have entirely disappeared. Indeed, in most cases of the disease, we do not find that the physical signs diminish as decidedly as the general symptoms." The author remarks that sometimes they even increase while the patient is gaining flesh, and his fever is considerably lessened. In these cases the deposition goes on, while the patient becomes less sensible to its effects, and thus even gains flesh. He (the author) therefore concludes that cod liver oil is not a specific against phthisis; it simply increases flesh, notwithstanding the disease; and sometimes may indirectly bring about a permanent cure in cases in which the tuberculous tendency has been ameliorated, and is therefore readily removed. He thinks, too, that cod liver oil will be more certainly useful in those cases in which patients are evidently disposed to consumption, and have inherited a strong tendency to it, although it is not yet developed. Under these circumstances, "where the patient is already thin or slightly emaciated, he directs it to be taken as an article of food rather than as a medicine." In these conclusions we entirely agree, as we have elsewhere in this number, it not yet having fallen to our lot to meet with more than improvement in the general health, in any case of decided phthisis in which we have used it. The following are the results obtained in the Pennsylvania Hospital during a trial of six months; the statement was prepared by Dr. Levick, one of the resident physicians:

"1st. *Of the Oil.*—That the light colored oil can be taken without difficulty by patients whose stomachs have steadily rejected the brown oil.

2d. *Of the mode of administration.*—That a few of the patients have taken the oil without any adjunct to disguise its taste. That its nauseating properties are corrected by its administration with milk; but that its taste is most effectually disguised by the froth of porter.

3d. *Of the time of its administration.*—That as a general rule it has been taken *before* meals, but that in four instances where it was not tolerated before meals it was readily taken after meals.

4th. *Of diarrhœa as a contraindication to its use.*—That the existence of diarrhœa is not a positive contraindication to its use. In three instances in which patients were thus affected, no increase of the symptoms was produced by its use, and no diminution by its abandonment. In a fourth instance, when the diarrhœa had previously existed, the discharge appeared to be increased by the exhibition of the oil, and abated with its withdrawal.

5th. *Of its effects in cases of phthisis pulmonalis.*—That patients using the oil have increased in flesh, in weight, and strength. That while using the oil, their cough and expectoration have diminished; that with some, hectic and rigors have entirely disappeared.

That six of them have been so much benefitted as to leave the hospital and resume their former occupations. That in one instance, a patient who entered the hospital with cough, copious purulent expectoration, extreme emaciation, inability to leave his bed, and with the physical signs of a cavity under the left clavicle, after six months use of the oil left the hospital weighing 140 lbs., with little or no cough, no hectic or rigors, and with an almost entire absence of expectoration: the physical signs having greatly diminished.

6th. *Of the physical signs.*—That the improvement of the physical signs is not coincident with that of the general symptoms.

7th. *Of its use in general scrofula.*—That in scrofulous diseases, where there was no reason to expect the existence of pulmonary tubercles, the improvement of the patient's health has been very decided.

8th. *Of congestion of lungs as produced by cod liver oil.*—That there has been no decided evidence of such a result following the use of the oil in the preceding cases. [Two patients of the twenty while using the oil had severe attacks of hæmoptysis, but there was no reason to refer them to the use of the remedy.]

9th. That in those cases which have terminated fatally, the appetite, the nutrition, and strength of the patient appeared for a time to be decidedly increased; that the life of the patient appeared to be in this manner temporarily protracted; but that for a few weeks immediately preceding death the remedy seemed to have entirely lost its value.

10th. *Length of time, &c.*—That to be of any decided permanent benefit its use must be steadily persisted in. It should be continued even after the most striking symptoms of the disease have in a great measure disappeared."

A short supplement on Spirometry concludes the work, a mode of exploration discovered by Dr. Hutchinson, and of which a brief notice was given in a review of Kirkes & Paget's Handbook of Physiology in this Journal, and which seems destined to throw light on the subject of physical diagnosis, even in cases where auscultation and percussion fail, as was seen in the case of Freeman, reported by Dr. Hutchinson, in which the Spirometer revealed disease not discoverable by the ordinary means of exploration. The profession owes a debt of gratitude to Dr. Gerhard for the excellent work with which he has presented them, a feeling which we hope will be substantially manifested in the rapid sale of the present edition.

